# Effect of the Particle Size and Matrix Strength on Strengthening and Damage Process of the Particle Reinforced Metal Matrix Composites

**DOI:** 10.3390/ma14030675

**Published:** 2021-02-01

**Authors:** Zhiyu Yang, Jianzhong Fan, Yanqiang Liu, Junhui Nie, Ziyue Yang, Yonglin Kang

**Affiliations:** 1 National Engineering & Technology Research Center for Non-Ferrous Metals Composites, GRINM Group Corporation Limited, Beijing 101407, China; yangzhiyu_grinm@163.com; 2 School of Materials Science and Engineering, University of Science and Technology Beijing, Beijing 100083, China; kangylin@ustb.edu.cn; 3 GRINM Metal Composites Technology Co. Ltd., Beijing 101407, China; liuyanqiang@grinm.com (Y.L.); niejunkey@163.com (J.N.); yangziyue@grinm.com (Z.Y.); 4 General Research Institute for Nonferrous Metals, Beijing 100088, China

**Keywords:** metal matrix composites (MMCs), in situ testing, strengthening mechanism, material design

## Abstract

Roles of the particle, strengthening, and weakening during deformation of the particle reinforced metal matrix composite, were studied using in situ technique. Composites with three different strengths Al-Cu-Mg alloy matrices reinforced by three sizes SiC particles were manufactured and subjected to in situ tensile testing. Based on in situ observation, damage process, fraction and size distribution of the cracked particles were collected to investigate the behavior of the particle during composite deformation. The presence of the particle strengthens the composite, while the particle cracking under high load weakens the composite. This strengthening to weakening transformation is controlled by the damage process of the particle and decided by the particle strength, size distribution, and the matrix flow behavior together. With a proper match of the particle and matrix, an effective strengthening can be obtained. Finally, the effective match range of the particle and the matrix was defined as a function of the particle size and the matrix strength.

## 1. Introduction

Particles reinforced metal matrix composites (PRMMCs), with its remarkable mechanical properties including high elastic modulus, specific strength, stiffness, good fatigue, and wear resistance, are attractive alternatives to lightweight applications in automobile and aviation industries [1,2,3,4]. To meet the needs for the development of advanced composites, many types of particles have been added into the metal matrices as reinforcements, such as oxides (e.g., Al_2_O_3_ [5,6]), nitrides (e.g., BN [7,8]), and carbides (e.g., B_4_C, TiC, SiC [9,10,11]). Among these reinforcements, the SiC particle with its combination of the high elastic modulus, high technical maturity, and low cost, has been one of the most commonly used reinforcements in industries [12]. However, the presence of the particle can reduce ductility, which limits the application in structural components. In addition, when the particle is matched with an improper matrix, the effect of the particle may transfer from strengthening to weakening [13,14,15]. To obtain a good strengthening results within a few experiments is still difficult.

In composites, the particles bear higher load than the matrix, which leads to a direct strengthening called the load transfer effect [16]. In addition, because of the difference on stiffness and thermal expansion of the particle and the matrix, additional residual stress and dislocation occur, which also strengthens the matrix [17,18]. Besides these, the particle itself blocks the dislocation during composite deformation and enhances the strain hardening of the composite [19]. On the other hand, the particle bears high load and cracks when the load on the particle exceeds its strength. This accumulating damage increases the probability of early fracture and reduces strength and ductility of the composite. Transition of these two opposite roles of the particle is controlled by damage process of the particle. The changing rule of this transition is key to the PRMMCs design.

Many efforts including experimental and numerical studies have been made on the strengthening effects and damage process of the particle (Table 1). Generally, the composite strength increases with the decreasing particle size [20,21,22], while the damage occurs continually during deformation and may lower the strength of the composite [23,24]. In composites, the actual strength of one particle is determined by its particle size, shape, and even the matrix strength. With larger particle size [21,25,26], larger aspect ratio [22,27] or higher matrix strength [28], damage of the particles increases. However, systemic data and analyses of the particle cracking process can hardly be found in the existing studies. Although the general rules of damage evolution are obtained under certain conditions, a systematic and comprehensive study is still needed. In numerical study, Weibull law is often used to modelling the damage behavior of the particle [29]. According to this law, particle cracks by its size order from larger size to small size in turn with the increasing stress, which does not entirely fit the experimental observation. In addition, because the main parameters are obtained by numeric fitting, it is hard to predict the strengthening result of the particle and matrix before the composite is manufactured and tested.

The aim of this article is to provide a better understanding on roles of the particle in strengthening of the composite. Damage process of the particle is fully observed and statistically analyzed. Effects of the particle size and the matrix strength on particle cracking process as well as their relations to the composite strength were studied. Based on these analyses, the effective match range was determined as a function of particle size and matrix strength. When developing new composites, this match range can predict the strengthening results of the composites before the materials are manufactured.

## 2. Materials and Methods

α-SiC particles with average sizes of 14.8, 19.6, and 29.9 μm were used as the reinforcements. Matrices were designed based on the Al-Cu-Mg alloy, which combined with good strength and elongation. By altering the amount of Cu and Mg, three matrices with different strengths were obtained. High pure powders of aluminum, magnesium, and copper were mixed according to the compositions shown in Table 2. Together with the 15 vol pct. of SiC particles, these powders were mixed in a ball miller with ball to powder weight ratio of 2:1 for 24 h. Then, the mixed powder is followed by the powder metallurgy process and extruded into 30 mm diameter rods at 520 °C. These rods were heat treated at 520 °C for 2 h, water quenched and aged at room temperature for at least 48 h before being shaped and tested. Three strengths matrices were reinforced by three sizes particles, respectively. They were named as a term of “matrix-particle size.” For example, “L-15” represents the L matrix reinforced by the SiCp of 14.8 μm particle.

The uniaxial tension specimens with a gauge length of 30 mm were tested on the AMSLER-100-20 universal testing machine (ZwickRoell, Kennesaw, GA, USA) at a strain rate of 0.5 mm/min. The elongation was measured using a clip-on extensometer. The yield strength is defined as 0.2% offset yield strength. At least 4 specimens of each composite were tested. The mechanical property used in this research was the average of 4 tests.

**Figure 1:**
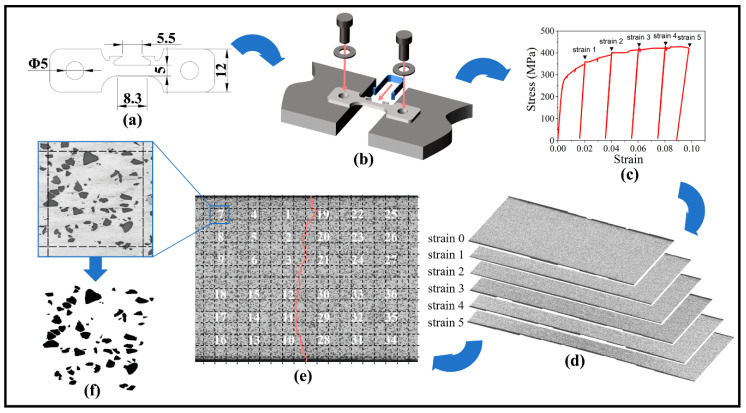
Schematic of the in situ observation process: (**a**) the dimension (mm) of the specimen, (**b**) the equipment setting, (**c**) the stress–strain curve of the intermittent loading testing, (**d**) the dimension of the image stacking, (**e**) the image sampling method, and (**f**) image of one sampling square and its binary image.

The equipment of the in situ tensile testing was provided by the CHINA UNITED TEST & CERTIFICATION CO., LTD., including a strain stage, which was equipped with an electric motor to generate load to the specimen, and a control program, which controlled and recorded the load and the displacement of the specimen. Flat tensile specimen, with a gauge length of 8.3 mm, width of 5 mm, and thickness of 0.9 mm, was used for in situ observation (Figure 1a). An extensometer was installed on the upper crabs to measure the strain of the gauge length. (Figure 1b). The flat side of the specimen was polished under standard procedure. The specimen was subjected to an intermittent uniaxial loading. First, it was strained to a given global strain of 0.01~0.05 at a strain rate of 4 μm/s and unloaded to 0 MPa state immediately. The value of the strain step depends on the elongation of the composite. A large strain step was used when the composite had a high elongation to ensure that at least five points were measured and it does not have too much interrupt points, which may increase accumulative error of the globe strain at the same time. Then, the full gauge length was photographed by the Zeiss Axiovert 200 MAT (manufactured by Carl Zeiss, Oberkochen, Badensko-wuertembersko, Germany) equipped with a high-resolution camera. Because the visual field of the microscopy is limited to cover full gauge length of the specimen at one time. These incomplete parts of the gauge length were joined together by the Photoshop software (Photoshop 2021, Adobe, San Jose, CA, USA) to obtain the whole picture of the gauge length of the specimen. After photographing, specimen was reloaded to a larger strain, unloaded, and photographed again. This procedure was repeated until the specimen was strain to failure (Figure 1c).

All images of the gauge length were stacked in the global strain order (Figure 1d) using Photoshop (Photoshop 2021, Adobe, San Jose, CA, USA). Then they were snapped and meshed with 200 μm squares. Three regularly spaced rows on both sides of the main crack were selected. In addition, images of six regularly spaced squares in these rows were taken into further analysis (Figure 1e). By increasing the contrast of these sampling images, the binarization images were obtain (Figure 1f). The number and shape information of the particle were collected from the binary images using Image-Pro Plus software (Image-Pro Plus 6, Media Cybernetics, Inc., Rockville, ML, USA).

## 3. Results

### 3.1. Mechanical Property

**Figure 2:**
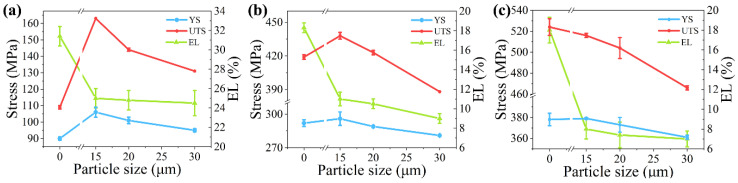
The yield strength (YS), ultimate strength (UTS), and elongation (EL) of the composites with (**a**) matrix L, (**b**) matrix M, and (**c**) matrix H. The mechanical properties of 0 μm particle represents the mechanical properties of the matrix.

The yield strength (YS), ultimate strength (UTS), and elongation (EL) obtained from the uniaxial tensile testing were shown in Figure 2. It can be seen that the composite strength increases with the increasing matrix strength. For 15 μm particle, the UTS of matrix (UTSm) increases from 109 MPa of L to 524 MPa of H and the UTS of composite (UTSc) increases from 163 MPa of L-15 to 516 MPa of H15. When particle size increases from 15 to 30 μm, both strength and the elongation of composites decrease. To be noted that strengths of some composites are lower than strengths of their matrices. For example, UTSc of M-20 are 423 MPa, which is lower than its UTSm of 419 MPa. It indicates that the effect of 20 μm particle on strength is transferred from strengthening to weakening on matrix M. The same phenomenon can be found in M-30, H-15, H-20, and H-30.

### 3.2. Particle Cracking

**Figure 3:**
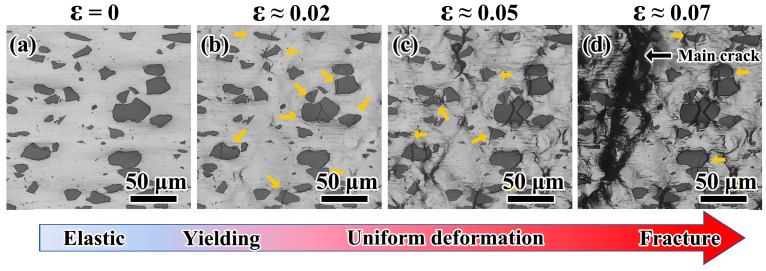
The typical damage evolution at the strain of (**a**) 0, (**b**) 0.02, (**c**) 0.05, and (**d**) 0.07 with a horizontal loading direction. The particles pointed by yellow arrows are the new cracked particle at their corresponding strain.

The in situ images from unloading state to final fracture were photographed. Based on these images, the characteristics of damage evolution were identified. Because the characteristics of damage evolution are similar in different composites, images of one typical composite are selected to show the damage process of the particle. Figure 3 shows the microstructure evolution of M-30 in a region near the main crack. Before composite yielding, no damage is found, and the microstructure remains as the initial state (Figure 3a). Almost all the deformation is elastic. After yielding, some particles, especially particles with large size, begin to crack and the surface relief is generated (Figure 3b). With stress and strain further increasing, particles with smaller size begin to crack and many slip bands are generated with an angle of 45° to the loading direction (Figure 3c). When the specimen is strained to its ultimate stress, only a few new cracked particles are found. With damage accumulating, the cracks of the particles connect and catastrophic fracture occurs (Figure 3d).

**Figure 4:**
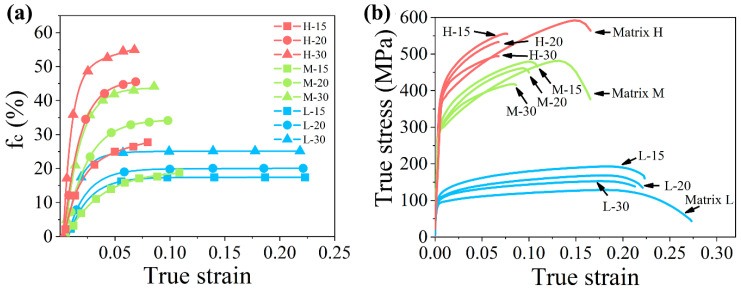
(**a**) The fraction of the cracked particle (fc) as a function of the true strain and (**b**) true stress–true strain curve of all composites and their matrices.

Fractions of the cracked particle fc were counted. Results are shown in Figure 4a. The true stress–true strain curves obtained from the uniaxial tension are shown in Figure 4b. It can be seen from Figure 4a that the start points of fc curves are close to their yield strains of the composites and raise rapidly with increasing strain. Most of the cracked particles crack within a strain of 0.06. With further deformation, the values of fc hardly increase. Comparing the different composites, fc increases with increasing particle size and matrix strength. Particle strength in the composite is related to not only its inherent strength but also its particle size and the matrix strength. Comparing the stress state shown in Figure 4b with the fc-strain curves in Figure 4a, a similar growth trend can be found. When the composite stress derives from its linear relation with the strain and transits to the plastic deformation stage, most of the particle cracking occurs.

**Figure 5:**
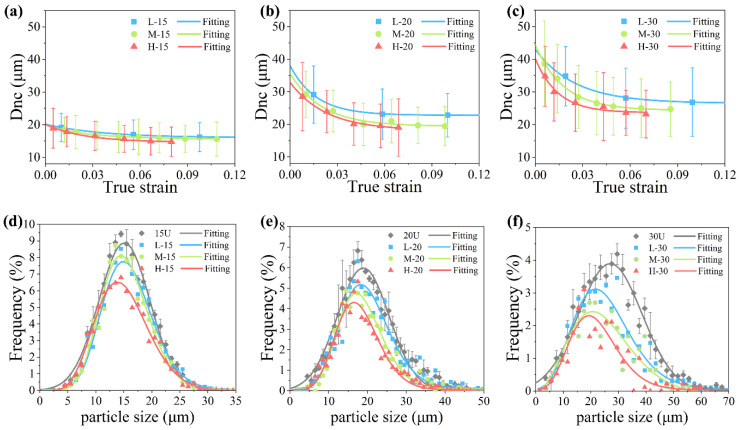
The diameter of new cracked particle (Dnc) in composites with particle sizes of (**a**) 15 μm, (**b**) 20 μm, and (**c**) 30 μm. The error bars of Dnc show the diameter deviations of Dnc in every strain step. As well as the size distribution of particles before loading (xxU) and their survived particles after fracture in composites with particle sizes of (**d**) 15 μm, (**e**) 20 μm, and (**f**) 30 μm. The error bars of the xxU show the frequency deviations in each particle size.

When the stress on the particle exceeds the Griffith criterion, particle cracking will occur. The critical fracture stress of the particle can be expressed as [30]:

σ_cra_ = K_p_/(r^0.5^) (1)

where K_p_ is a constant and related to the fracture toughness of the particle. The smaller size particle has a higher fracture stress. It can also mean that a smaller size of the new cracked particle in one strain indicates a higher load on the particle at the same time. The average diameter of the new cracked particle (Dnc) in one strain step can exhibit the stress level of the particle and the results are shown in Figure 5a–c. It can be seen that the Dnc values of all composites decrease with the increasing strain, which means the particle bears an increasing load during composite deformation. The Dnc values decrease more and more slowly with the increasing strain. In the later stage of the composite deformation, the slowly increasing load on the particle and the high strength of the survived small particle reach a balance, i.e., the particle stop cracking. When the larger size particle or higher strength matrix is used, the particle cracking will continue until this balance is reached in a larger strain, which causes a large amount of particle cracking and a reduction in the mechanical properties.

The particle size distribution before loading and after fracture obtained from the in situ images is shown in Figure 5d–f. It can be found that the frequency of the survive particle after fracture decrease and the peaks of the distribution shift to the relatively smaller size, which means more larger size particle in one size distribution cracked. Moreover, the higher of the matrix strength is, the smaller of the average size of the survived particle will be. The average size of 15, 20, and 30 μm particles decrease to 14.3 μm, 17.2, and 20.5 μm after fracture. The particle smaller than this size can cause an effective strengthening and less damage on composite.

### 3.3. Crack Propagation

**Figure 6:**
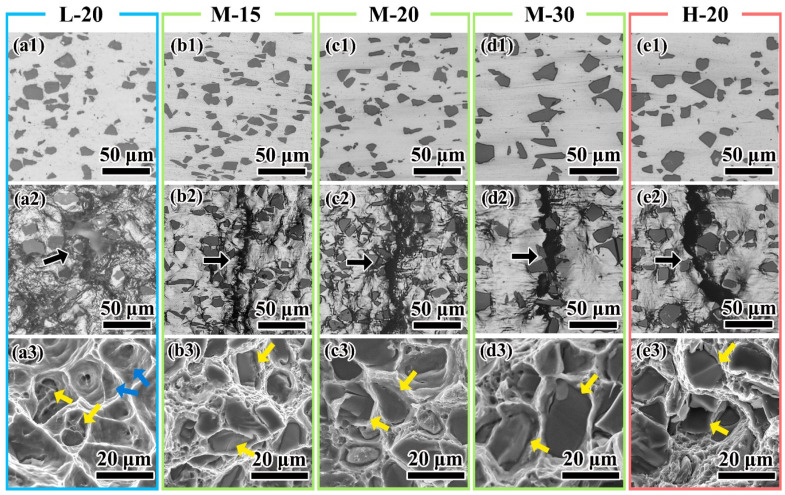
Crack propagation and fracture morphology of (**a**) L-20, (**b**) M-15, (**c**) M-20, (**d**) M-30, and (**e**) H-20. The state of mages in every groups are (1) before loading, (2) after fracture in the region in which the main crack (pointed by arrow) passes through, and (3) the fracture morphology of the main crack.

In low strength composite, such as L-20, the low flow strength of the matrix lowers the fraction of the cracked particle. The main crack occurs in the matrix and is caused mainly by matrix-induced damage, such as void coalescence (Figure 6(a2)). The fracture surface is mainly composed of large dimples (Figure 6(a3) pointed by blue arrows) and decohesion at interface between the matrix and the particle are also found (Figure 6(a3) pointed by yellow arrows). As matrix strength and particle size increase, more particles begin to crack and the propagation mode of the main crack changes from mainly growing through the matrix (Figure 6(b2,c2)) to straight crossing the particle (Figure 6(d2,e2) pointed by yellow arrows). The proportion of the cracked particle on the fracture surface increases (Figure 6(b2–e2)). These phenomena indicate that the fracture mechanism changes from matrix-induced damage to particle-induced damage.

## 4. Discussion

Because little particle cracking occurs when composite yielding, the influence of the particle cracking on the yield strength is small. However, after yielding, the particles begin to crack to release the concentrated stress. This accumulating damage reduces the strain hardening effect of the particles, rises the probability of fracture and leads to a reduction in the UTSc. The cracking process transfers the roles of the particle from strengthening to weakening. This process is affected by not only the particle itself but also the matrix. Only with a proper match of the particles and the matrices, an effective strengthening on the composite can be achieved.

**Figure 7:**
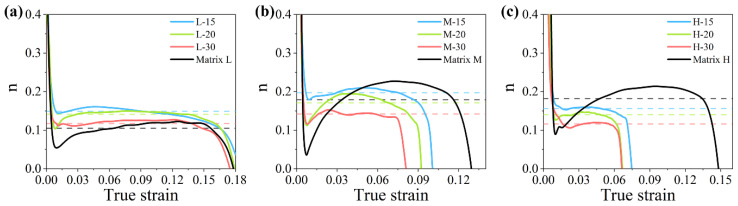
The relation n(ε) between the strain hardening component n and true strain of composites. the results of n(ε) of composites using (**a**) matrix L, (**b**) matrix M, and (**c**) matrix H are shown together with the n(ε) of their matrices. The value of the dash line is the average of n value in deformation stage of the composite in same color, and these n values are shown in Table 3.

The true stress–true strain relation can be expressed by Hollomon equation [31] as:

σ = Kε^n^ (2)

where σ is the true stress, ε is the true strain, and K and n are two constants, which are called as the strain hardening coefficient and the strain hardening exponent, respectively. The value of n can represent the strain hardening rate of the composite. With a large n value, the composite will have a higher strain hardening rate. The relation of n with the true strain n(ε) can also be obtained by a derivation of the Equation (2) as:

n(ε) = (ε/σ) × (dε/dσ)(3)

where the dε/dσ is the slope of the true stress–true strain curve of the composite and the strain hardening exponent n is the average of the liner stage of the n(ε) in a high strain level. The relations n(ε) calculated from the true stress–true strain curves shown in Figure 4b are shown in Figure 7, and the calculating results of n are shown in Table 3. As shown in Figure 7a, strain hardening rate of the composites has a little decrease with the increasing strain. The deformation remains stable, though a little of damage occurs in the composite with low strength matrix. However, when the matrix strength increases, the deformation of the composite decreases earlier compared to its matrix (Figure 7b,c). This is because the amount of the broken particles increases in a high strength matrix composite, and it can lead the deformation into unstable state when the amount of the broken particle exceeds the limit of the matrix. Composites with high strength matrix fracture earlier and can no longer get strengthened from the strain hardening.

**Figure 8:**
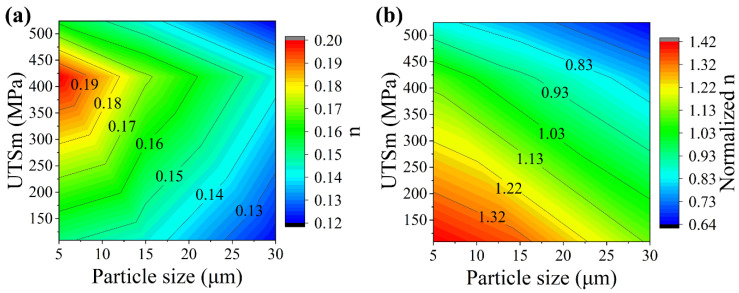
The relationship of (**a**) strain hardening exponent “n” and (**b**) normalized n (normalized to its n of matrix) of composites with the matrix ultimate strength (UTSm) and the particle size.

To better reveal the variation of n value with the particle size and the matrix stress, Figure 8a was constructed based on the results in Table 3 in interpolation method. As shown in Figure 8a, n value decreases with the increasing particle size when the matrix strength is given. However, it has a maximum value with a moderate strength matrix when the particle size is given. The strengthening effect of the particle on strain hardening is resulted of the competition between the strain hardening rate of the matrix and the damage growth rate of the composite. Thus, it is influenced by both particle and the matrix. In matrix with a low strain hardening rate, matrix storages limited dislocations and causes damage of cavities, which results in the limit strengthening effect of the particle. In matrix with a high strain hardening rate, the high stress during deformation causes large amount of particle cracking. Too much cracked particles would lead to an early fracture before the matrix can obtain enough strengthening from the strain hardening. This also results in low strengthening effect. Thus, the particle needs a proper match of a matrix to balance the strain hardening rate and the damage growth rate to achieve the best strengthening effect. In this research, when the matrix with UTSm approximately of 419 MPa is used, the particle has the best strengthening effect.

By dividing the n of the composite to that of its matrix, the normalized n can be obtained. The normalized n can be seen as the strengthening ability of the particle to one matrix on the strain hardening and results are shown in Table 4. Based on these results, Figure 8b was constructed in interpolation method to show the relationship of the normalized n with the particle size and the UTSm.

It can be seen from Figure 8b that the normalized n decreases with the increasing particle size and UTSm. When it decreases below the value of 1, the particle can no longer strengthen the matrix and begin to weaken the matrix due to the large quantity of damage occurred in composites. Polynomial was used to fit the relationship of normalized n with UTSm and particle size:

N = a − bD − cσ + dD^2^ − eσ^2^ + fDσ (4)

where N is the Normalized n, D is the particle size, and σ is the UTSm. The fitting results are shown in Table 5. Based on the Equation (4), the effective matching results of the particles and the matrices can be calculated by selecting a proper matrix strength and particle size to obtain a N value over 1. For the particles and the matrices used in this research, the largest particle sizes to obtain effective strengthening for matrix L, M, and H are 37 μm, 18, and 9 μm, respectively. The highest matrix strengths for particle sizes of 15 μm, 20 μm, and 30 μm to have an effective strengthening are 455 MPa, 399 MPa, and 259 MPa, respectively.

## 5. Conclusions

In this study, effect of the particle size and the matrix strength on strengthening and damage process of the particle reinforced metal matrix composites was investigated using in situ testing. Damage evolutions of 9 combinations of SiCp/Al composites were collected and characterized in terms of the fraction of the cracked particles, the diameter of the new increased cracked particles, and the size distribution of the survived particles. Based on the results, following conclusions can be drawn:

(1) The larger size particles tend to crack earlier during deformation of the composite. Use of the particle with small average size and narrow size range can decrease the particle-induced damage and obtain a good strengthening effect on the composites.
(2) Matrix strength also influences the particle cracking. With improper match of the high-strength matrix to the particle, large amount of particle cracking will occur. Too much particle-induced damage will reduce the strengthening effect of the particle and change the role of the particle from strengthening to even weakening.
(3) The effective match range of the particle and the matrix is determined as the relation of the normalized strain hardening exponent n with the matrix strength and the particle size. The combination of the particle and the matrix should have an N value over 1 to obtain an effective strengthening. For the particles and the matrices used in this article, the largest particle sizes to obtain effective strengthening for matrix L, M, and H are 37 μm, 18 μm and 9 μm, respectively. The highest matrix strengths for particle size of 15, 20, and 30 μm to have an effective strengthening are 455 MPa, 399 MPa, and 259 MPa, respectively.

## Figures and Tables

**Table 1:** Research methods of strengthening and damage process in particles reinforced metal matrix composites (PRMMCs).

	Strengthening Effects on			Damage Process
Methods in	Yield Strength	Ultimate Strength	Elongation	
Existing research	T (20–22), IS (28), N (30)	T (20,28), IS (28), N (27)	T (22), IS (28)	ST (23,24), IS (25,26), N (29)
This article	T, IS	T, IS	T, IS	IS

T = tensile testing, IS = in situ testing, N = numerical study, ST = synchrotron tomography.

**Table 2:** Chemical compositions of the matrices.

Matrix	Chemical Compositions (wt.%)		
	Cu	Mg	Al
L	–	–	100
M	2.6	1.0	Bal
H	4.2	1.6	Bal

**Table 3:** The strain hardening components n of composites and their matrices.

Materials	L	L-15	L-20	L-30	M	M-15	M-20	M-30	H	H-15	H-20	H-30
n values	0.105	0.149	0.141	0.117	0.179	0.197	0.171	0.142	0.182	0.156	0.140	0.116

**Table 4:** The normalized strain hardening components n of composites.

Materials	L-15	L-20	L-30	M-15	M-20	M-30	H-15	H-20	H-30
Normalized n	1.419	1.343	1.114	1.101	0.948	0.782	0.857	0.769	0.637

**Table 5:** Fitting results of the relation of normalized n with the ultimate strength of matrix (UTSm) and the particle size.

a	b	c	d	e	f	R-Square
1.94	−3.29 × 10^−2^	−5.11 × 10^−4^	2.23 × 10^−4^	−1.64 × 10^−6^	1.17 × 10^−5^	0.99

## Data Availability

Data is contained within the article.
